# Adaptive coloration in pied flycatchers (*Ficedula hypoleuca*)—The devil is in the detail

**DOI:** 10.1002/ece3.7048

**Published:** 2021-01-24

**Authors:** Päivi M. Sirkiä, Anna Qvarnström

**Affiliations:** ^1^ Finnish Museum of Natural History Zoology Unit University of Helsinki Helsinki Finland; ^2^ Department of Ecology and Genetics Animal Ecology Uppsala University Uppsala Sweden

**Keywords:** *Ficedula hypoleuca*, melanin coloration, ornaments, pied flycatcher, plumage coloration, sexual selection

## Abstract

Understanding the origin and persistence of phenotypic variation within and among populations is a major goal in evolutionary biology. However, the eagerness to find unadulterated explanatory models in combination with difficulties in publishing replicated studies may lead to severe underestimations of the complexity of selection patterns acting in nature. One striking example is variation in plumage coloration in birds, where the default adaptive explanation often is that brightly colored individuals signal superior quality across environmental conditions and therefore always should be favored by directional mate choice. Here, we review studies on the proximate determination and adaptive function of coloration traits in male pied flycatchers (*Ficedula hypoleuca*). From numerous studies, we can conclude that the dark male color phenotype is adapted to a typical northern climate and functions as a dominance signal in male–male competition over nesting sites, and that the browner phenotypes are favored by relaxed intraspecific competition with more dominant male collared flycatchers (*Ficedula albicollis*) in areas where the two species co‐occur. However, the role of avoidance of hybridization in driving character displacement in plumage between these two species may not be as important as initially thought. The direction of female choice on male coloration in pied flycatchers is not simply as opposite in direction in sympatry and allopatry as traditionally expected, but varies also in relation to additional contexts such as climate variation. While some of the heterogeneity in the observed relationships between coloration and fitness probably indicate type 1 errors, we strongly argue that environmental heterogeneity and context‐dependent selection play important roles in explaining plumage color variation in this species, which probably also is the case in many other species studied in less detail.

## Graphical abstract

We review numerous studies on both proximate and ultimate factors behind extensive plumage color variation in male pied flycatchers and particularly zoom in on the most unknown questions and suggest future research avenues. There is strong evidence that interspecific interactions with collared flycatchers are of crucial importance for explaining the origin and maintenance of plumage color variation in male pied flycatchers, but the role of avoidance of hybridization in driving character displacement in plumage between the two flycatcher species may not be as important as initially thought. Our review suggests that plumage traits should not be expected to signal high quality as such but rather different male competitive strategies, and a main take‐home message is that selection patterns acting on signaling traits are more diverse and fluctuating than generally expected.

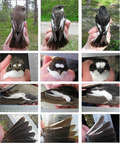

## 1 INTRODUCTION

Explaining how phenotypic variation emerges and is maintained in nature is major goal in evolutionary biology. Hypotheses are tested and improved, thereby facilitating movement toward general principles (see Box 1). However, the eagerness to find unadulterated explanatory models may sometimes lead to severe underestimations of the complexity of selection patterns acting in nature. Stringent hypothesis testing requires stringent design of experiments and data collection. Capturing the complexity of natural processes therefore requires enormous efforts of detailed empirical data collection across various environmental conditions. With the fast development of sequencing and other omics methods, the bottleneck for hypothesis testing is in many fields of biology shifting from data collection to analysis (McPherson, 2009). This technology‐driven massive production of data down at the molecular resolution also from natural populations will revolutionize the field of evolutionary biology (Husby et al., 2019). However, the analyses aiming at detecting signals of selection at the genomic level are still blunt with respect to disentangling selection processes from population demographic processes and fined‐scaled patterns of fluctuating selection cannot be reconstructed based on genomic data alone. Detailed studies on the behavior and ecology of model species may therefore experience a revival when the aim is to understand processes that maintain heritable phenotypic variation in populations and how this, in turn, affects the evolutionary potential of populations to respond to a rapidly changing climate and ecosystems.

**How genetic variation in fitness traits can be maintained in natural populations?**

In general, genetic variation in phenotypic traits that are linked to fitness is expected to be eroded by natural and sexual selection (Fisher, 1930). Broad explanations for the persistence of variation in fitness‐related traits are, for example, mutation–selection balance (Rowe & Houle, 1996), fluctuating selection (Bell, 2010; Cornwallis & Uller, 2010), negative frequency‐dependent selection (Fisher, 1930; Fitzpatrick et al., 2007), and genic capture model for sexually selected traits (Tomkins et al., 2004). In mutation–selection balance, the question is whether mutations can generate new variation as quickly as it is eroded by selection (Tomkins et al., 2010). Fluctuating selection can maintain variation because the performance of different types of individuals varies across environmental conditions in time and space—a type advantageous in one environment may not be optimal in another. Negative frequency‐dependent selection selects for rare phenotypes and thus increases a population's genetic variance. Genic capture model suggests that male display traits are costly to produce and hence depend upon overall condition, which itself is dependent upon genes at many loci (Tomkins et al., 2004), and thus, sexually selected traits capture genetic variation in all traits that influence individual condition.

In birds, plumage coloration is often strikingly variable, but the processes promoting the maintenance of this variation are surprisingly poorly understood. Both the intensity of coloration and pattern formed by different colors can vary. Color itself is produced either by different pigments or by structure. Further, different types of coloration differ in many ways, such as the extent of genetically determined variance (Hill & McGraw, 2006). The most common form of pigmentation in birds is that caused by melanins, which yield various black, brown, gray, and rufous colors. There are two categories of melanin pigments: eumelanin, conferring dark black or brown hues, and pheomelanin, conferring reddish‐brown hues (McGraw, 2006). In addition to colors produced by pigments in the tissue, nonpigmented feathers in combination with pigmented ones also form striking plumage patterns, such as bars, spots, and different patches that vary, for example, in size or frequency. In addition to pigmented and depigmented coloration, structural coloration at near‐ultraviolet wavelengths (UV‐A; 320–400 nm) is visible to birds (Cuthill et al., 2000).

Plumage coloration has many adaptive functions, and it is used in intraspecific signaling in a number of ways: to convey information about variation on individual as to quality, Fisherian attractiveness, behavioral strategies, genetic compatibility, kinship, individual identity, and presence (Dale, 2006). Coloration thus plays a major role in many social contexts, and bright coloration and various adornments in animals are mostly assumed to have evolved through sexual selection (Andersson, 1994; Darwin, 1871; Hill, 2006b). However, coloration may have many additional adaptive functions, such as vision enhancement, protection from abrasion, bacterial degradation, or predation avoidance (Bortolotti, 2006).

The vast majority of the studies on the function and evolution of plumage coloration in birds have focused on female choice based on male coloration, benefits to females from assessing male color, or color displays as signals of aggression and dominance (Andersson, 1994; Hill & McGraw, 2006). Different possible adaptive functions of plumage coloration have generally been studied in different species making it difficult to evaluate the relative importance of the different processes. Detailed behavioral studies of individual species under various natural conditions can therefore improve our general understanding on the relative importance of various processes and whether and how the overall selection patterns of plumage coloration fluctuate. A species that for a long time has been the focus of numerous behavioral and ecological studies is the pied flycatcher. This is a small insectivorous passerine with highly variable male plumage coloration. Plumage coloration of male pied flycatchers is one of the most studied examples of color variation in birds (Tables 1 and 2), but the results about the processes that have the potential to maintain color variation within and among populations have not been summarized before. Most of this research has focused on the dorsal black‐brown melanin coloration and white forehead patch, but has recently been further expanded to also cover the white wing and tail patches and ultraviolet reflectance. Here, we review studies on both proximate and ultimate factors behind color variation in male pied flycatchers and particularly zoom in on the most unknown questions, such as the role of survival, density‐dependent and sexually antagonistic selection on male coloration, and suggest future research avenues. We attempted to locate all scientific papers published in English, which assess the function or characteristics of male plumage color in the pied flycatcher using experimental or observational methods published prior to March 2020 by searching the ISI Web of Science database. We used the search terms “pied flycatcher” and “*Ficedula hypoleuca*.” In addition to publications found in the described search, we also examined whether citations in the found publications included further relevant information for our review. In addition to the scientific papers found, we have used older literature and scientific literature in Russian and in German.

**Table 1:** Studies reported on the selection acting on male plumage coloration traits in the pied flycatcher

Male plumage trait	Variable^a^	Life history trait	Trait	Data^b^, ^c^	Field/aviary	Effect	Selection toward	Direction of selection dependent on	Authors
Black‐brown dorsal coloration	cq	Female mate choice	Gaining EPP	corr	F	No			Lehtonen, Primmer, et al. (2009)
	cq	Female mate choice	Gaining EPP	corr	F	Yes	Black		Canal et al. (2011)
	cq	Female mate choice	Losing paternity	corr	F	No			Moreno et al. (2010)
	cq	Female mate choice	Losing paternity	corr	F	No			Moreno, Martínez, et al. (2013)
	cq	Female mate choice	Losing paternity	corr	F	No			Rätti et al. (1995)
	cq	Female mate choice	Laying date	corr	F	Yes	Black		Røskaft and Järvi (1983)
	cq	Female mate choice	Laying date	corr	F	No			Potti and Montalvo (1991a)
	cq	Female mate choice	Laying date	corr	F	Yes		Space	Kerimov et al. (1994)
	cq	Female mate choice	Laying date	corr	F	Yes	Black		Gálvan and Moreno (2009)
	cq	Female mate choice	Laying date	corr	F	Yes	Black		Järvi et al. (1987)
	cq	Female mate choice	Losing paternity	corr	F	Yes	Brown		Lifjeld et al. (1997)
	cq	Female mate choice	Losing paternity	corr	F	No			Lehtonen, Primmer, et al. (2009)
	cq	Female mate choice	Pairing success	corr	F	No			Alatalo et al. (1984)
	cq	Female mate choice	Pairing success	exp	F	No			Alatalo et al. (1986)
	cq	Female mate choice	Pairing success	corr	F	No			Slagsvold (1986)
	cq	Female mate choice	Pairing success	corr	F	Yes	Black		Järvi et al. (1987)
	cq	Female mate choice	Pairing success	corr	F	Yes		Time, among seasons	Lifjeld and Slagsvold (1988)
	cq	Female mate choice	Pairing success	corr	F	No			Alatalo et al. (1990)
	cq	Female mate choice	Pairing success	exp	F	No			Alatalo et al. (1990)
	cq	Female mate choice	Pairing success	exp, cm	F	No			Alatalo et al. (1990)
	cq	Female mate choice	Pairing success	exp	A	No			Alatalo et al. (1990)
	cq	Female mate choice	Pairing success	corr	F	No			Dale and Slagsvold (1990)
	cq	Female mate choice	Pairing success	corr	F	No			Potti and Montalvo (1991a)
	cq	Female mate choice	Pairing success	corr	F	Yes	Black		Dale and Slagsvold (1996)
	cq	Female mate choice	Pairing success	corr	F	Yes	Black		Sætre et al. (1994)
	cq	Female mate choice	Pairing success	corr, exp, cm	A	Yes	Black		Sætre et al. (1994)
	cq	Female mate choice	Pairing success	corr	F	No			Sirkiä and Laaksonen (2009)
	cq	Female mate choice	Pairing success	exp, cm	A	Yes		Sympatry/allopatry	Sætre et al. (1997)
	cq	Female mate choice	Polygamy	exp	A	Yes	Black	Trade‐off with pairing status	Slagsvold and Drevon (1999)
	cq	Female mate choice	Polygamy	corr	F	Yes	Black		Von Haartman (1985)
	cq	Female mate choice	Polygamy	corr	F	No			Lundberg and Alatalo (1992)
	cq	Breeding success	Brood mass	corr	F	Yes	Black		Sætre et al. (1995)
	cq	Breeding success	Clutch size	corr	F	No			Røskaft and Järvi (1983)
	cq	Breeding success	Clutch size	corr	F	Yes		Space	Kerimov et al. (1994)
	cq	Breeding success	Clutch size	corr	F	Yes		Age	Gálvan and Moreno (2009)
	cq	Breeding success	Clutch size	corr	F	Yes		Env. conditions	Sirkiä et al. (2010)
	cq	Breeding success	Nestling body condition	corr	F	No			Gálvan and Moreno (2009)
	cq	Breeding success	Nestling mass	corr	F	No			Slagsvold and Lifjeld (1988)
	cq	Breeding success	Nestling mass	corr	F	Yes	Black		Røskaft and Järvi (1983)
	cq	Breeding success	Nestling mass	corr	F	Yes	Black		Järvi et al. (1987)
	cq	Breeding success	Nestling mass	exp	F	Yes		Env. conditions	Järvistö (2015)
	cq	Breeding success	Nestling mortality	corr	F	Yes		Env. conditions	Sirkiä et al. (2010)
	cq	Breeding success	Number of nestlings	corr	F	No			Røskaft and Järvi (1983)
	cq	Breeding success	Number of fledglings	corr	F	Yes		Env. conditions	Sirkiä et al. (2010)
	cq	Breeding success	Number of recruits	corr	F	Yes		Age	Alatalo et al. (1994)
	cq	Breeding success	Polygyny	corr	F	Yes		Pop. mean coloration	Røskaft et al. (1986)
	cq	Life‐time breeding output	Number of fledglings	corr	F	Yes		Breeding history	Ivankina et al. (2001)
	cq	Longevity	Predation risk	corr	F	Yes	Brown		Slagsvold et al. (1995)
	cq	Longevity	Return rate	corr	F	No			Lundberg and Alatalo (1992)
	cq	Longevity	Return rate	corr	F	Yes	Brown		Røskaft et al. (1986)
	cq	Longevity	Return rate	corr	F	Yes	Brown		Järvi et al. (1987)
	cq	Longevity	Return rate	corr	F	No			Slagsvold and Lifjeld (1988)
	cq	Longevity	Return rate	corr	F	No			Alatalo et al. (1994)
	cq	Longevity	Return rate	corr	F	No			Ivankina et al. (2001)
	cq	Longevity	Return rate	corr	F	Yes	Black		Potti and Montalvo (1991b)
	cq	Longevity	Return rate	corr	F	Yes	Black		Belskii and Lyakhov (2004)
UV reflectance	cq	Female mate choice	Gaining EPP	corr	F	No			Lehtonen, Primmer, et al. (2009)
	cq	Female mate choice	Losing paternity	corr	F	Yes	High UV		Lehtonen, Primmer, et al. (2009)
	cq	Female mate choice	Nest building	exp, cm	A	Yes	High UV		Siitari et al. (2002)
	cq	Female mate choice	Pairing success	exp, cm	F	Yes		Time, within season	Sirkiä and Laaksonen (2009)
	cq	Female mate choice	Pairing success	corr	F	Yes		Melanin coloration	Sirkiä and Laaksonen (2009)
Forehead patch	ps	Female mate choice	Gaining EPP	corr	F	No			Lehtonen, Primmer, et al. (2009)
	ps	Female mate choice	Gaining EPP	corr	F	Yes	Larger patch		Canal et al. (2011)
	ps	Female mate choice	Laying date	corr	F	No			Potti and Montalvo (1991a)
	ps	Female mate choice	Laying date	corr	F	No			Gálvan and Moreno (2009)
	ps	Female mate choice	Laying date	corr	F	Yes		Env. conditions	Sirkiä et al. (2010)
	ps	Female mate choice	Losing paternity	corr	F	No			Lehtonen, Primmer, et al. (2009)
	ps	Female mate choice	Losing paternity	corr	F	No			Moreno et al. (2010)
	ps	Female mate choice	Losing paternity	corr	F	No			Moreno, Martínez, et al. (2013)
	ps	Female mate choice	Pairing success	corr	F	Yes	Larger patch		Potti and Montalvo (1991a)
	ps	Female mate choice	Pairing success	corr	F	No			Dale et al. (1999)
	ps	Female mate choice	Pairing success	exp, cm	F	No			Dale et al. (1999)
	ps	Female mate choice	Pairing success	corr	F	No			Sirkiä and Laaksonen (2009)
	ps	Breeding success	Egg volume	exp, cm	F	Yes	Larger patch		Osorno et al., 2006
	ps	Breeding success	Clutch size	corr	F	Yes		Age	Gálvan and Moreno (2009)
	ps	Breeding success	Nestling body condition	corr	F	No			Gálvan and Moreno (2009)
	ps	Breeding success	Nestling size	exp, cm	F	Yes	Smaller patch		Sanz (2001)
	ps	Longevity	Return rate	corr	F	No			Dale et al. (1999)
Wing patch size	ps	Female mate choice	Losing paternity	corr	F	No			Moreno et al. (2010)
	ps	Female mate choice	Losing paternity	corr	F	No			Moreno, Martínez, et al. (2013)
	ps	Female mate choice	Pairing success	corr	F	Yes	Larger patch		Sirkiä and Laaksonen (2009)
	ps	Breeding success	Nestling mortality	corr	F	Yes		Env. conditions	Sirkiä et al. (2010)
	ps	Breeding success	Fledgling production	corr	F	Yes		Env. conditions	Teerikorpi et al. (2018)
Tail patch size	ps	Female mate choice	Pairing success	corr	F	No			Sirkiä and Laaksonen (2009)

^a^ Coloration trait measured, cq = color quality, ps = patch size.

^b^ corr = correlational, exp = experimental.

^c^ cm = experimental color manipulation.

**Table 2:** Studied associations between male plumage traits and habitat, behavioral, morphological, and physiological traits

Male plumage trait	Variable^a^		Trait in question	Data^b^, ^c^	Field/aviary	Effect	Association dependent on	Reference
Black‐brown dorsal coloration	cq	Environment	Breeding habitat	corr	F	No		Belskii and Lyakhov (2004)
	cq	Environment	Breeding habitat	corr	F	Yes		Ivankina et al. (1995)
	cq	Environment	Singing microhabitat	corr	F	Yes		Ivankina et al. (1995)
	cq	Behavior	Territorial behavior	corr	F	Yes		Slagsvold and Lifjeld (1988)
	cq	Behavior	Aggression	corr	F	Yes		Järvi et al. (1987)
	cq	Behavior	Aggression	exp, cm	F	Yes		Huhta and Alatalo (1993)
	cq	Behavior	Aggression	exp, cm	F	No		Huhta and Alatalo (1993)
	cq	Behavior	Aggression	corr	F	No		Huhta and Alatalo (1993)
	cq	Behavior	Aggression	corr	F	No		Breiehagen and Saetre (1992)
	cq	Behavior	Fearfulness	corr	F	Yes		Camacho et al. (2018)
	cq	Behavior	Natal dispersal	corr	F	Yes		Camacho et al. (2018)
	cq	Behavior	Timing of arrival	corr	F	Yes	Study year	Slagsvold and Lifjeld (1988)
	cq	Behavior	Timing of arrival	corr	F	No		Siitari and Huhta (2002)
	cq	Behavior	Nestling feeding rate	corr	F	No		Järvistö (2015)
	cq	Behavior	Nestling feeding rate	corr	F	No		Slagsvold and Lifjeld (1988)
	cq	Behavior	Nestling feeding rate	corr	F	Yes		Saetre et al. (1996)
	cq	Behavior	Nestling feeding rate	corr	F	Yes		Sætre et al. (1997)
	cq	Behavior	Singing activity	corr	F	Yes	Weather	Ilyina and Ivankina (2001)
	cq	Behavior	Song versatility	corr	F	Yes		Lampe and Espmark (1994)
	cq	Behavior	Timing of molting	corr	F	No		Slagsvold and Lifjeld (1988)
	cq	Endoparasitism	Blood parasites	corr	F	Yes		Dale et al. (1996)
	cq	Endoparasitism	Blood parasites	corr	F	No		Dale et al. (1996)
	cq	Morphology	Body mass	corr	F	No		Slagsvold and Lifjeld (1988)
	cq	Morphology	Body mass	corr	F	No		Belskii and Lyakhov (2004)
	cq	Morphology	Wing length	corr	F	Yes		Belskii and Lyakhov (2004)
	cq	Physiology	Immune response	corr	A	Yes		Ruuskanen et al. (2013)
	cq	Physiology	Immune response	corr	A	No		Ruuskanen et al. (2013)
	cq	Physiology	Immuno response	corr	F	Yes	Molting stage	Kerimov et al. (2012)
	cq	Physiology	Immuno response	exp	F	Yes	Molting stage	Kerimov et al. (2018)
	cq	Physiology	Immuno response	exp	F	No		Kerimov et al. (2018)
	cq	Physiology	Metabolic rate	corr	A	No		Ruuskanen et al. (2013)
	cq	Physiology	Metabolic rate	corr	F	Yes		Røskaft et al. (1986)
	cq	Physiology	Metabolic rate	corr	F	Yes		Kerimov et al. (2014)
	cq	Physiology	Metabolic rate	corr	F	Yes		Gavrilov et al. (1993)
	cq	Physiology	Oxidative stress	corr	F	No		Lopez‐Arrabe et al. (2014)
	cq	Physiology	Oxidative stress	corr	F	No		Lopez‐Arrabe et al. (2014)
	cq	Physiology	Oxidative stress	corr	F	No		Lopez‐Arrabe et al. (2014)
	cq	Physiology	Oxidative stress	corr	F	Yes		Moreno et al. (2011)
	cq	Physiology	Oxidative stress	corr	F	Yes	Temperature	Teerikorpi et al. (2019)
	cq	Physiology	Sperm morphology	corr	F	Yes		Calhim et al. (2009)
Forehead patch	ps	Behavior	Aggression	exp	A	Yes		Järvistö et al. (2013)
	ps	Behavior	Breeding dispersal	exp, cm	F	Yes		Sanz (2001)
	ps	Behavior	Nestling feeding rate	corr	F	No		Dale et al. (1999)
	ps	Behavior	Nestling feeding rate	exp, cm	F	Yes		Sanz (2001)
	ps	Behavior	Territorial behavior	exp, cm	F	Yes		Osorno et al. (2006)
	ps	Behavior	Territorial behavior	exp	A	No		Dale et al. (1999)
	ps	Endoparasitism	Blood parasites	corr	F	No		Potti and Merino (1996)
	ps	Endoparasitism	Blood parasites	corr	F	No		Dale et al. (1999)
	ps	Physiology	Oxidative stress	corr	F	No		Moreno et al. (2011)
	ps	Physiology	Metabolic rate	corr	A	No		Ruuskanen et al. (2013)
	ps	Physiology	Immune response	corr	A	No		Ruuskanen et al. (2013)
	ps	Physiology	Stress	corr	F	Yes		Lobato et al. (2010)
	ps	Physiology	Immune response	corr	A	No		Ruuskanen et al. (2013)
	ps	Physiology	Oxidative stress	corr	F	No		Lopez‐Arrabe et al. (2014)
	ps	Physiology	Oxidative stress	corr	F	No		Lopez‐Arrabe et al. (2014)
	ps	Physiology	Oxidative stress	corr	F	No		Lopez‐Arrabe et al. (2014)
	ps	Physiology	Oxidative stress	corr	F	No		Moreno et al. (2011)
UV reflection	cq	Behavior	Timing of arrival	corr	F	Yes		Siitari and Huhta (2002)
	cq	Physiology	Metabolic rate	corr	A	No		Ruuskanen et al. (2013)
	cq	Physiology	Immune response	corr	A	Yes		Ruuskanen et al. (2013)
	cq	Physiology	Immune response	corr	A	No		Ruuskanen et al. (2013)
Wing patch size	ps	Physiology	Oxidative stress	corr	F	No		Moreno et al. (2011)
	ps	Physiology	Metabolic rate	corr	A	No		Ruuskanen et al. (2013)
	ps	Physiology	Immune response	corr	A	No		Ruuskanen et al. (2013)
	ps	Physiology	Immune response	corr	A	No		Ruuskanen et al. (2013)
	ps	Physiology	Oxidative stress	corr	F	Yes		Lopez‐Arrabe et al. (2014)
	ps	Physiology	Oxidative stress	corr	F	No		Lopez‐Arrabe et al. (2014)
	ps	Physiology	Oxidative stress	corr	F	Yes		Lopez‐Arrabe et al. (2014)
	ps	Physiology	Oxidative stress	corr	F	No		Moreno et al. (2011)

^a^ Coloration trait measured, cq = color quality, ps = patch size.

^b^ corr = correlational, exp = experimental.

^c^ cm = experimental color manipulation.

## 2 THE PIED FLYCATCHER

**FIGURE 1:**
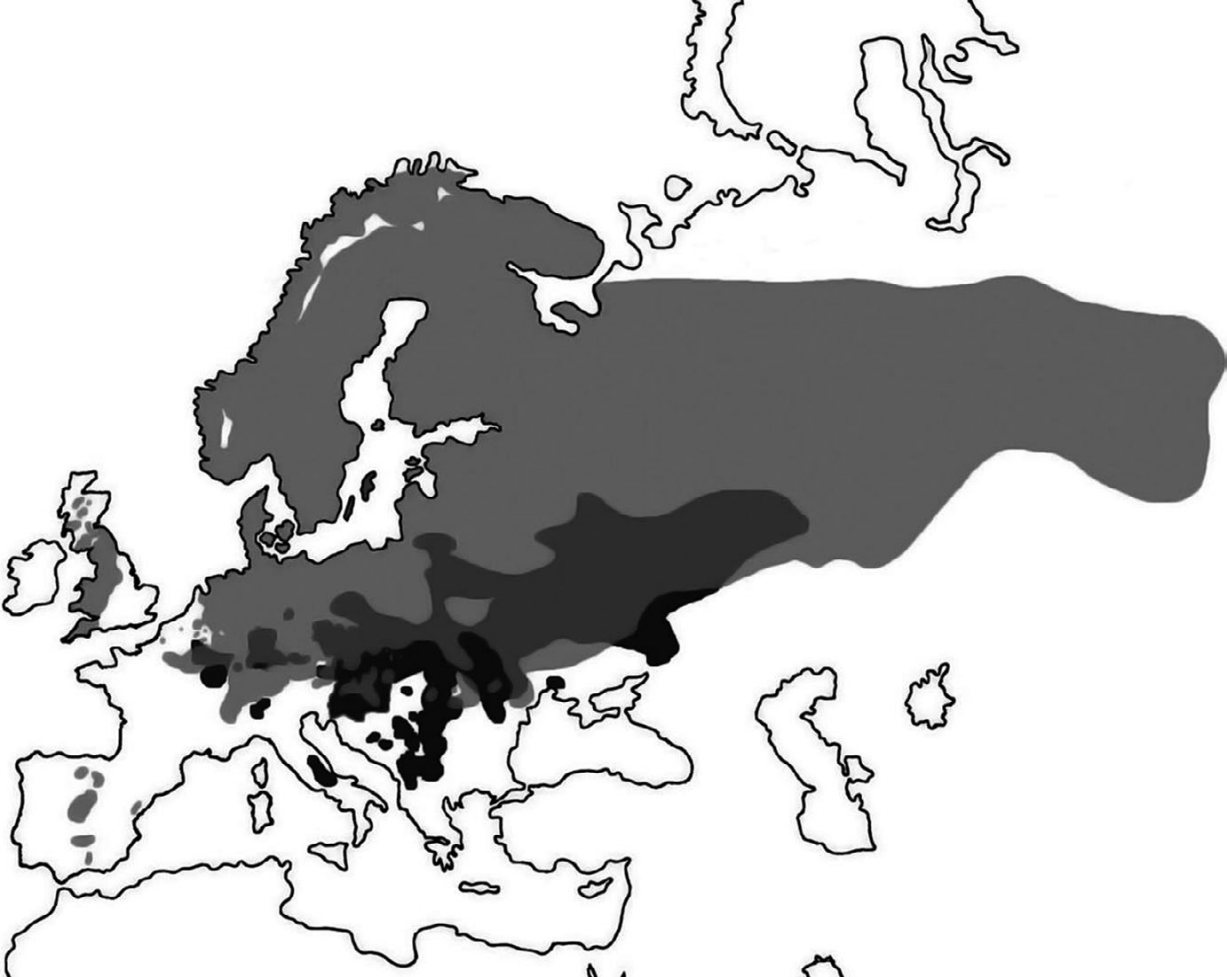
Breeding range of the pied flycatcher and the closely related sister species collared flycatcher. Light gray represents the breeding range of the pied flycatcher alone, medium gray the sympatric breeding area of both the pied and the collared flycatcher, dark gray the breeding area of the collared flycatcher alone. Map after Laaksonen et al. (2015), originally modified from Birds of the Western Palearctic (Cramp & Simmons, 2006) and Flint et al. (1984)

**FIGURE 2:**
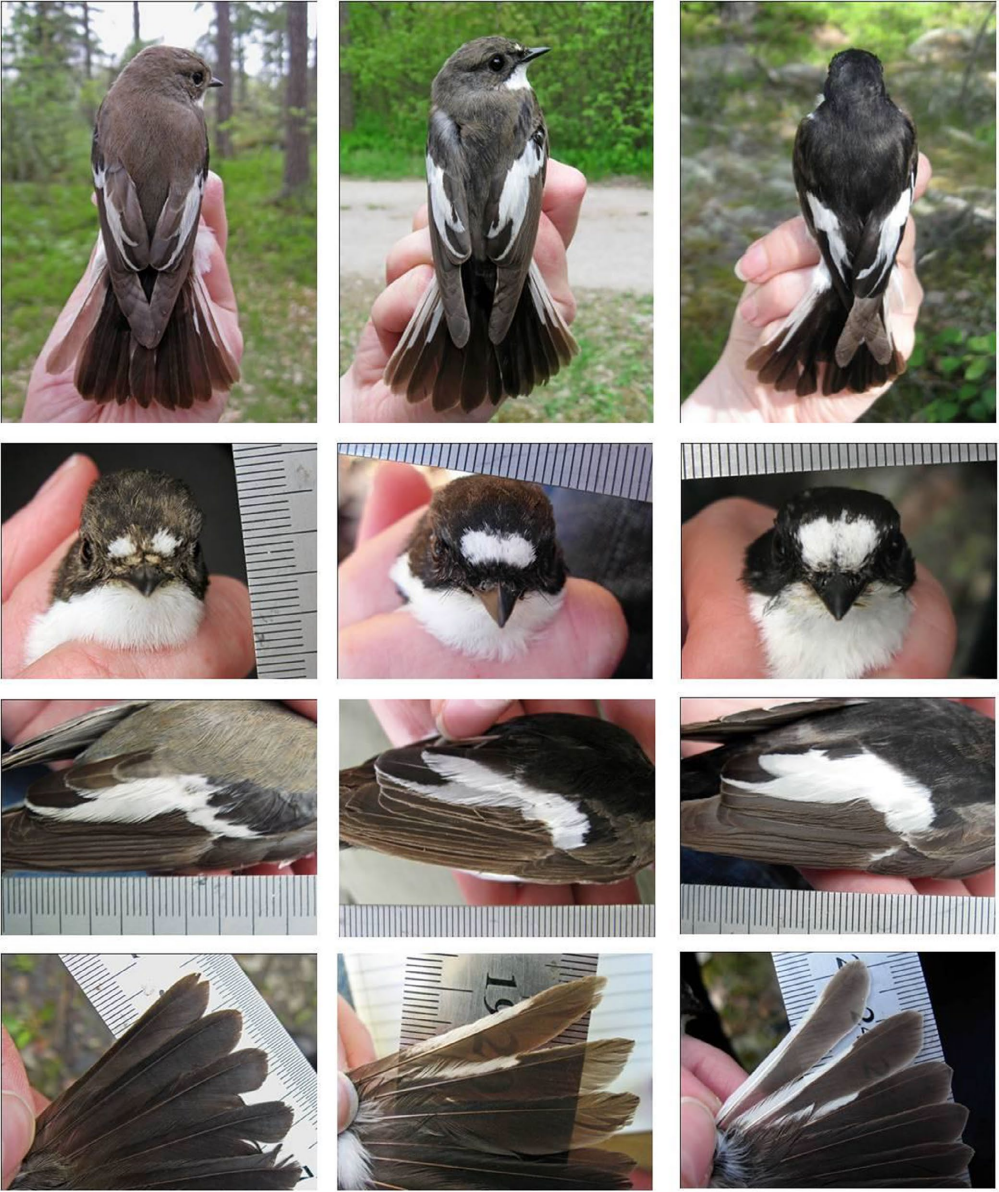
Some examples of males with different dorsal coloration, forehead, wing, and tail patches photographed in southern Finland. Within‐population variation in the most variable populations represents substantial proportion of among‐population variation

The pied flycatcher is a hole‐nesting passerine accepting nest boxes for breeding. This characteristic together with the wide breeding range (Figure 1) has made the pied flycatcher a popular model species in numerous ecological and evolutionary studies (reviewed by Lundberg & Alatalo, 1992; e.g., Sætre et al., 1997; Both et al., 2006; Vallin et al., 2012; Ahola et al., 2007). The pied flycatcher breeds from Europe to western Siberia and winters in sub‐Saharan Africa. While variation in female and nestling plumage coloration is limited, male plumage coloration is highly variable with respect to dorsal black‐brown coloration, UV reflectance, and sizes of white patches on forehead, wing, and tail (Figure 2, e.g., Laaksonen et al., 2015). Dorsal black‐brown coloration and forehead patch size have traditionally been the most studied male traits in the pied flycatcher, but recently also other traits, UV reflectance of plumage, and white patches on wing and tail have received more attention. In Central European sympatric areas with the collared flycatcher (Figure 1), most male pied flycatchers are brownish with small white ornamental patches, whereas in allopatric areas, the male phenotype is highly variable. In allopatric areas, the frequency of more conspicuous males, with darker dorsal coloration, higher reflectance in UV, larger forehead and wing patches, and smaller tail patches, increases with the distance to the sympatric Central European contact zone (Laaksonen et al., 2015). There is however extensive variation both between individuals and between years in mean phenotype in allopatry (Laaksonen et al., 2015; Sirkiä et al., 2013).

While the occurrence of different color types of pied flycatcher males has been described since the 18th century (Lundberg & Alatalo, 1992), Drost (1936) was the first to examine color variation in this species more in detail. The numbers of studies on pied flycatcher coloration raised markedly during the 1980s and 90s, and the field has been active since then. Before the 21st century, the studies were mostly restricted to the dorsal black‐brown coloration. Studies on coloration traits have been conducted almost throughout the whole breeding range of the species (Figure 1), but many studies have been conducted in Fennoscandia, Spain, and Cental Europe with a particular focus on areas where the pied flycatcher co‐occur with the collared flycatcher (Figure 1).

## 3 PROXIMATE DETERMINATION OF COLOR VARIATION

Revealing the proximate sources of within‐population variation in coloration is an important key prerequisite for understanding the evolution of various color traits. This is because the degree of genetic variance determines whether and how quickly there can be an evolutionary response to selection acting on particular traits. Apart from partitioning variance in coloration into the genetic and environmental components, further dissection of the environmental components (and also into possible genetic‐by‐environmental interactions in the determination of a trait) can reveal important information about the potential signaling functions of the trait (e.g., whether variation in the trait reveals information about the current state and condition of the bearer to potential competitors and mates).

There is covariation between plumage traits and several behavior, physiological, and morphological traits in male pied flycatchers (Table 2), suggesting that these plumage traits may function as signals in intraspecific communication (see further in *Hypothesis for adaptive functions of color variation* below). Honest signaling requires that there is a cost associated with the signal (Grafen, 1990; Maynard Smith & Harper, 1988). There may be a cost of producing the coloration trait, costs of maintenance or displaying it, or a cost arising through a shared biochemical pathway of producing the coloration trait and another important fitness trait such as immune function. Overall, there are few experimental studies on vertebrates on the causal effects of body condition on coloration traits, except from the relatively well‐understood carotenoid‐based coloration (reviewed by Hill & McGraw, 2006), and the results are restricted to a limited number of species especially in the case of melanin coloration (Roulin, 2016). Understanding the relative importance and interaction between different factors, such as variation in genes, body condition, and diet, in determining plumage coloration is demanding as feathers are inert structures and replaced normally only a few times in the life cycle of an individual bird. The few existing experimental studies on condition‐dependent expression of coloration traits in pied flycatchers are limited to maternal effects (Ruuskanen et al., 2013) and experimentally activated immune defense (Kilpimaa et al., 2004) (see Table 1). The melanin colored dorsal plumage and the white forehead patches are molted in wintering areas in Africa. White wing patches are constituted by white patches on tertials, also molted in Africa, and white bands on flight feathers, molted in the breeding range during late breeding or after breeding. White patches in tail feathers of adults males are formed during late breeding or soon after breeding, while yearling birds do not molt their tail feathers (Lundberg & Alatalo, 1992; Svensson, 1992). Thus, a general limiting factor for understanding sources of condition‐dependent variation in plumage coloration of male pied flycatchers is there are, to our knowledge, no experimental studies performed at the wintering areas where these traits are mainly produced.

### 3.1 Black‐brown dorsal coloration

The most conspicuously variable coloration trait in male pied flycatchers is the eumelanin‐based dorsal coloration that ranges from completely brown to black (Drost, 1936; Lundberg & Alatalo, 1992). Melanins (in contrast with carotenoids) are endogenously produced, and variation in their formation and deposition are known to be mainly genetically based (McGraw, 2006; Roulin, 2004, 2016; Roulin & Ducrest, 2013). The dorsal black‐brown coloration in pied flycatcher is indeed highly heritable (reported h2 values varying from 0.6 to 0.88) (Alatalo et al., 1994; Grinkov, 2000; Lehtonen, Laaksonen, et al., 2009). Recent whole‐genome sequencing efforts (e.g., Ellegren et al., 2012) are providing promising background for understanding of the genetic determination of plumage coloration traits in *Ficedula* flycatchers. There have also been efforts to find candidate genes for melanin and structural coloration (Lehtonen et al., 2011), but detailed knowledge on the underlying genomic basis of these traits remains mainly open. However, general difficulties in detecting quantitative trait loci in natural populations are still limiting our abilities to reveal specific loci associated with color trait variation (Kardos et al., 2016).

There is some evidence suggesting that that melanin coloration can be physiologically and energetically costly (Griffith et al., 2006; Jawor & Breitwisch, 2003) and therefore affected by the current state of individuals and by environmental conditions experienced (Griffith et al., 1999; Horth, 2006; Järvistö et al., 2016; Lepetz et al., 2009; Roulin, 2016; Roulin, Almasi, et al., 2008). Old pied flycatcher males tend to be slightly darker than young ones: A modest (ca 20%, i.e., one Drost score) change occurs between the ages of one and two years (Lundberg & Alatalo, 1992). Males have been observed to become darker after dry and windy compared with moist and less windy wintering conditions (Järvistö et al., 2016).

Pleiotropic effects of the complex melanocortin system is generally known to cause covariation between the degree of melanin‐based coloration morphology, physiology, behavior, or reproductive traits in pied flycatchers and in other species (Ducrest et al., 2008; Roulin, 2004, 2016). Covariations between dorsal coloration and physiological traits such as oxidative stress, metabolic rate, sperm quality, and immune response have been reported (see Table 2). Relationships between metabolic rate and melanin coloration have been suggested to signal male phenotypes adapted to different environmental conditions, while correlations between sperm morphology and immune response suggest that melanin coloration may indicate variation in male quality or reproductive tactic. Several lines of evidence suggest covariation between the degree of melanin coloration and behavioral traits such as aggression, fearfulness, nestling feeding rate, and natal dispersal (see Table 2). It is likely that different reported covariations arise due to shared biochemical pathway of producing the melanin coloration and a correlated trait in question. It is however notable that often results of the studied relationships reported are conflicting (see above, Table 2), suggesting potentially context dependence of these relationships.

### 3.2 White ornamental patches

In male pied flycatchers, conspicuous white patches on the forehead, wing, and tail all vary greatly in size and shape. Most males have a white forehead patch, the size of which has a heritable component (Potti & Canal, 2011, but see Dale et al., 1999). Forehead patch size is highly repeatable across years (*r* = .72) (Järvistö et al., 2016) indicating high heritability or a permanent environmental effect on trait expression. Older males have slightly larger forehead patch size than young males (Gálvan & Moreno, 2009; Järvistö et al., 2016), and in addition, an Iberian population very old males tended to reduce their forehead patches (Moreno et al., 2019). Environmental variables experienced during the molting period on the wintering grounds do not predict within‐individual variation of the trait (Järvistö et al., 2016). There is some evidence that the trait is signaling early‐life individual quality (Dale et al., 1999) and that costs of breeding, that is, unfavorable conditions during breeding and relatively early timing of breeding are followed by forehead patch decrements in males (Moreno et al., 2019). The experimental activation of immune defense reduced the expression of male forehead patch size compared with the control males within the same season (Kilpimaa et al., 2004), indicating that immune defense costs may trade‐off with the maintenance costs of a white forehead patch. While overall production costs of nonpigmented plumage traits can be questionable, depigmented white patches are costlier to maintain than dark areas of the feathers as bacterial degradability of unmelanized white areas in feathers is higher than melanized parts of the same feathers (Ruiz‐De‐Castaneda et al., 2012). Related to production of the trait, most studies on relationships between physiological traits and forehead patch expression have failed to detect any covariation (Table 2) and it remains open whether forehead patch size signals variation in individual condition or health in pied flycatchers. Experiments in the closely related collared flycatcher suggest that social costs of cheating enforce honest signaling of male fighting ability in this species, which vary little in their black dorsal coloration (Pärt & Qvarnström, 1997; Qvarnström, 1997). The signaling function of the white forehead patch in pied flycatchers, in relation to variation in dorsal coloration, remains an open question and may vary between different populations.

The size of the white wing patch is moderately repeatable across years (*r* = .34), and older males have larger wing patches than young males (Järvistö et al., 2016). Environmental conditions experienced during prebreeding molt in wintering areas have been found to affect wing patch size. During dry wintering conditions, male wing patch decreases within individuals and large‐patched individuals have a higher return rate than small‐patched ones (Järvistö et al., 2016). This suggests that after dry nonbreeding conditions, large wing patch size can be a more informative indicator of male quality than during other years as only individuals with good condition or resources have been able to produce large with patches in such conditions. Opposite to wintering conditions, weather conditions experienced during breeding have not been observed to be associated with between‐years within‐individual changes in the size of the wing patches (Moreno et al., 2019). Some of the results on the potential covariation between wing patch size and oxidative stress suggest that the trait could signal individual quality (Lopez‐Arrabe et al., 2014, see Table 2). In vitro tests showed that early‐breeding males have lower bacterial degradability of the white wing patches compared with males breeding late in the season (Ruiz‐De‐Castaneda et al., 2015), suggesting that white wing patches may indicate feather and individual quality.

The role of tail patch size of males has received very little attention. The expression of the trait is dependent on sex and age so that males have less white on their outer retrixes than female, and in males, the trait is less pronounced in older males than in young males (Belskii, 2006). The proximate determination of the expression of the trait remains open.

### 3.3 UV reflectance of the white wing patch

There is some evidence that UV reflectance in bird feathers is both heritable (Johnsen et al., 2003; Py et al., 2006) and condition‐dependent (reviewed by Hill, 2006a; Keyser & Hill, 1999). UV reflectance is sometimes considered as a quality measure of given coloration trait. In the pied flycatcher, most of the studies on UV reflectance have focused on the white wing patch. In the pied flycatcher, within‐individual repeatability of UV reflectance is moderate (*r* = .46) and the trait has not been found to be affected by environmental factors during prebreeding molting on the wintering grounds (Järvistö et al., 2016). Older males have higher UV reflectance than young males (Järvistö et al., 2016). Few studies have so far investigated covariation between UV reflectance (Table 2) and other traits, and overall, the proximate determination of the trait remains poorly understood. In addition to UV reflectance of white wing patch, some studies have investigated UV reflectance of the whole upper parts of male plumage (Siitari et al., 2002) and crown, mantle, and breast (Siitari & Huhta, 2002).

Overall, different male coloration traits are interconnected (Laaksonen et al., 2015). In particular, correlations between plumage darkness and UV reflectance of white wing patch and forehead, wing, and tail patch sizes are moderate to strong suggesting that these traits coevolve. There are, however, differences in which factors affect the expression of the plumage traits. Black‐brown dorsal coloration and forehead patch size are highly repeatable across years, while repeatability of the size and UV reflectance of the white wing patch is weaker. In addition, environmental conditions experienced during prebreeding molt affect wing patch size. Such differences suggest that different plumage traits can convey different types of information about the males.

## 4 HYPOTHESES FOR ADAPTIVE FUNCTIONS OF COLOR VARIATION

There have been numerous studies performed on the adaptive functions and mechanisms that may explain variation of plumage coloration in pied flycatchers. Most of these studies have focused on the melanin‐based black‐brown dorsal coloration, and several hypotheses regarding possible adaptive functions of this striking variation have been proposed (Lundberg & Alatalo, 1992; Røskaft et al., 1986; Sirkiä et al., 2010; Table 3). Both natural selection and sexual selection have been suggested to act on dorsal coloration, but the reported relationships between melanin coloration and various fitness components such as sexual attractiveness, breeding success, and longevity are diverse (Table 1, see below). When it comes to associated fitness advantages, other coloration traits than degree of melanin coloration have received markedly less attention. Below, we concentrate on reported tests of proposed adaptive functions of dorsal color variation (Table 3). The role of other plumage traits is discussed when it is applicable.

**Table 3:** Suggested hypotheses for adaptive function in dorsal black‐brown coloration in male pied flycatchers

Adaptive function	Hypothesis		Suggested by	Support for the hypothesis	No support for or against the hypothesis
Strategies to cope with biotic and abiotic environments	Temperature	Color types are adaptations to different temperatures	Ilyina and Ivankina (2001)	Ilyina and Ivankina (2001), Sirkiä et al. (2010), Järvistö (2015)	
	Humidity	Color types are adaptations to different amounts of precipitation	Sirkiä et al. (2010)	Sirkiä et al. (2010)	
	Habitat	Color types are adaptations to different habitats	Ivankina et al. (1995)	Ivankina et al. (1995)	Belskii and Lyakhov (2004)
	Predation	Unprofitable prey: conspicuous males avoided by prey	Røskaft et al. (1986)	Götmark (1992, 1993, 1995), Post and Götmark (2006)	Slagsvold et al. (1995)
		Conspicuous prey more easily discovered by prey	Von Haartman (1985)	Slagsvold et al. (1995)	Götmark (1992, 1993, 1995), Post and Götmark (2006)
	Parasitism	Color types are adaptations to differences in parasitism	Dale et al. (1999)		Dale et al. (1999)
Signaling function in communication with conspecifics	Dominance signaling	Delayed plumage maturation	Slagsvold and Lifjeld (1988)		Curio (1959), Alatalo et al. (1982), Harvey et al. (1985), Slagsvold and Lifjeld (1988)
		Conspecific female mimicry	Slagsvold and Lifjeld (1988)	Sætre et al. (1993), Saetre and Slagsvold (1996)	Slagsvold and Lifjeld (1988), Lundberg and Alatalo (1992), Calhim et al. (2014)
		Signaling of presence	Slagsvold and Lifjeld (1988)	Dale and Slagsvold (1996)	Huhta and Alatalo (1993)
		Status signaling	Slagsvold and Lifjeld (1988)	Järvi et al. (1987), Slagsvold and Lifjeld (1988), Slagsvold and Sætre (1991)	Breiehagen and Saetre (1992), Huhta and Alatalo (1993)
	Coloration is signaling male quality to females	Black males have higher pairing/breeding success than brown males (in all contexts)	Røskaft and Järvi (1983)	Røskaft and Järvi (1983), Von Haartman (1985), Järvi et al. (1987), Sætre et al. (1994, 1995), Dale and Slagsvold (1996), Lifjeld et al. (1997), Gálvan and Moreno (2009), Canal et al. (2011)	Røskaft and Järvi (1983), Alatalo et al. (1984), Alatalo et al. (1986), Alatalo et al. (1990), Alatalo et al. (1994), Dale and Slagsvold (1990), Slagsvold (1986), Slagsvold and Lifjeld (1988), Lifjeld and Slagsvold (1988), Potti and Montalvo (1991a), Lundberg and Alatalo (1992), Kerimov et al. (1994), Rätti et al. (1995), Sætre et al. (1997), Slagsvold and Drevon (1999), Ivankina et al. (2001), Lehtonen, Primmer, et al. (2009), Gálvan and Moreno (2009), Sirkiä and Laaksonen (2009), Sirkiä et al. (2010), Moreno et al. (2010), Moreno, Velando, et al. (2013), Järvistö (2015)
Signaling function in communication with heterospecifics	Avoidance of hybridization	Avoiding hybridization with female collared flycatchers	Røskaft et al. (1986)	Sætre et al. (1993); Sætre et al. (1997), Veen et al. (2010)	Vallin et al. (2012)
	Heterospecific female mimicry	Avoiding competition with male collared flycatchers	Røskaft et al. (1986)	Král et al. (1988), Vallin et al. (2012)	

### 4.1 Strategies to cope with biotic and abiotic environments

The sign and magnitude of natural selection on melanin coloration often seems to depend on ecological or environmental factors (Antoniazza et al., 2010; Roulin et al., 2011), suggesting that different melanin phenotypes are adapted to different conditions (e.g., Almasi et al., 2008; Ducrest et al., 2008; Roulin et al., 2008). Variation in temperature, humidity, habitat, predation, and parasitism has been suggested to drive variation in plumage coloration within and among populations of pied flycatchers.

#### 4.1.1 Temperature

Several studies support the hypothesis that the different melanin color types observed among male pied flycatchers are adapted to different prevailing temperatures (Table 1). Black males seem to be more active and experience high reproductive performance during cold springs (Ilyina & Ivankina, 2001; Sirkiä et al., 2010). Higher activity during pairing may lead to access to more resources provided for female or differences in female investment. Environment‐dependent selection on male dorsal coloration is however parallel between life history phases: The reproductive output of black males is highest when it is cold during the egg‐laying but warm during the nestling period (Sirkiä et al., 2010). Nestlings of dark males are lighter and have higher mortality in relatively low temperatures during the nestling period (Järvistö, 2015; Sirkiä et al., 2010). It has been shown experimentally that it is the melanin coloration of the foster parent and not the genetic parent that matters during the rearing period (Järvistö, 2015). Further, it has been shown that foster offspring of black males seem to suffer from oxidative stress under relatively cold weather compared with those of brown males (Teerikorpi et al., 2019). Taken together, these results show that temperature‐dependent variation in reproductive success mainly is explained by differences in parental behaviors, which in turn are associated with variation in melanin coloration (Järvistö, 2015; Sirkiä et al., 2010; Teerikorpi et al., 2019). These findings are compatible with the idea of pleiotropic effects of genes regulating the synthesis of melanins being the key links between climatic adaptations and eumelanin‐based plumage coloration (Roulin, 2004). In the pied flycatcher, there is also temperature‐dependent selection on forehead patch size so that females paired with males with large forehead patch (while other plumage traits being controlled for) start laying eggs earlier in springs with low temperature (Sirkiä et al., 2010), which indicates a context‐dependent success in either intrasexual competition or mate choice. While dorsal coloration and forehead patch size are moderately correlated (Laaksonen et al., 2015), it may however be that the traits convey different types of information about the males.

In addition to pleiotropic effects of genes, melanin coloration may play a significant role, for example, in thermoregulation (Dreiss et al., 2016; McGraw, 2006; Roulin, 2004) or in feather structure (Bonser, 1995; Koskenpato et al., 2016). Melanin coloration has potentially significant effects on the heat balance in small birds, and dark plumage is known to absorb more solar radiation than light plumage (Wolf & Walsberg, 2000). In the pied flycatcher, potential benefits of thermoregulation properties or feather structure of differently colored plumage remain unstudied.

#### 4.1.2 Humidity

In addition to temperature, rainfall and humidity are suggested to be conditions to which different color phenotypes are adapted. According to the ecogeographic Gloger's rule, birds in areas of high relative humidity are darker than those living in areas of dry climate (Burtt & Ichida, 2004; Zink & Ramsen, 1986). There are many potential reasons for why dark coloration could be favored in areas of high humidity such as differential bacterial degradation (Burtt & Ichida, 2004), background matching (Zink & Ramsen, 1986), and enhanced drying (Burtt, 1981). In the pied flycatcher, the breeding success of different melanin phenotypes is not dependent on the amount of precipitation (Sirkiä et al., 2010). Instead, selection on wing patch size has been found to be dependent on amount of precipitation during breeding season so that the nestlings of males with large wing patches have lower mortality in years with high levels of rainfall compared to the males with small wing patch (Sirkiä et al., 2010). Further, Teerikorpi et al. (2018) showed that after experiencing a relatively dry winter, large‐patched males were more successful in attracting females that laid large clutches and were more likely to survive, while the opposite was true after moist winters. Interestingly, this phenomenon led to a difference in fledgling numbers between differently colored males only during years with dry winters and high precipitation during the breeding season.

#### 4.1.3 Predation and parasitism

Cryptic coloration is an important source of protection from predation in birds and other organisms (e.g., Bortolotti, 2006). It has been also suggested that the exposure to predation risk can modify antipredator behavior in relation to sexual coloration (Møller et al., 2011). Von Haartman (1985) suggested that more conspicuous dark male pied flycatchers would be more easily discovered by predators. This hypothesis is supported by the fact that right after breeding season, males molt their conspicuous breeding plumages to cryptic and females have rather cryptic plumages throughout the year. An opposite view was suggested by Røskaft et al. (1986) who proposed that conspicuous males could be avoided by the predators, and thus, in populations with higher predation pressure males would be on average darker. Dark males had higher probability to disappear during breeding season than brown males (Slagsvold et al., 1995), suggesting that conspicuous males would have higher predation rate. Sparrow hawks (*Accipiter nisus*) were more likely to attack stuffed females than males during breeding (Götmark, 1995; Post & Götmark, 2006) and on migration (Götmark, 1992, 1993), which does not support the hypothesis that wearing dull plumage would be an antipredator strategy. Detectability may be habitat‐dependent as at least human observers find males more conspicuous than females against the ground, but detectability does not differ against trees (Götmark & Hohlfält, 1995). One must however bear in mind that in light of current knowledge, pied flycatchers are able to distinguish brown males from intraspecific females (Calhim et al., 2014), and thus, the experiments comparing female and male coloration cannot be directly interpreted to apply to male coloration. A further complicating factor is that predation risk often depends on behavior and differences in behavior related to coloration (e.g., Da Silva et al., 2013). Differences in behavior, for example, in the openness of singing posts of males with different degrees of melanin coloration (Ivankina et al., 1995) could lead to differential predation rates of male color types in the pied flycatcher. It also remains unclear whether densities of avian predators are associated with male pied flycatcher plumage coloration among populations as suggested by Von Haartman (1985).

Several studies on a number on taxa, including birds, have shown a link between melanin coloration and differential parasite loads (e.g., Chakarov et al., 2008; Galeotti & Sacchi, 2003; Jacquin et al., 2011; Lei et al., 2013), suggesting that parasitism could play a crucial role in selection on coloration traits in natural populations. In the pied flycatcher, a handful of studies have investigated the relationships between parasite load and male color phenotypes (Table 1). Overall, potential selection acting on coloration via both endo‐ and ectoparasite load remains mostly undiscovered in the pied flycatcher. In addition, melanin plumage coloration may also play a significant role, for example, in microbial resistance (Burtt & Ichida, 2004; Goldstein et al., 2004) and in protection from wear (Delhey et al., 2010; Ward et al., 2002), but these potentially adaptive functions remain unstudied in the pied flycatcher (but see Ruiz‐De‐Castaneda et al., 2012).

#### 4.1.4 Habitat

With respect to their coloration, individuals are often nonrandomly distributed among habitats (Roulin, 2004; Zink & Ramsen, 1986). In the pied flycatcher, male melanin phenotypes occur in the same habitats (Belskii & Lyakhov, 2004; Lundberg & Alatalo, 1992). However, within a habitat type Ivankina et al. (1995) found that darker males prefer more open breeding microhabitat and were singing in more open locations than dull brown males, which suggests that melanin color phenotypes may be adapted to different microhabitats. Potential habitat‐dependent success of different phenotypes remains unexplored. It also remains unstudied whether different male phenotypes have different abilities to cope with stress caused by asynchrony with the habitat‐dependent insect food availability (Burger et al., 2012; Sirkiä et al., 2018; Veen et al., 2010) and whether such differences can lead to differences in habitat‐dependent success among the color morphs.

#### 4.1.5 Environment‐dependent selection maintaining color variation

If variation in coloration traits is assumed to signal variation in individual quality across environmental conditions, the lack of consistent fitness effects may be interpreted as type I errors as in recent meta‐analyses on other species (Parker, 2013; Sánchez‐Tójar et al., 2018). The alternative interpretation is that variation in fitness‐related traits is subject to highly fluctuating selection patterns in which performance of different genotypes varies across contexts in both time and space. Altogether, 21 of 84 measures that have been used to estimate selection on coloration traits were found to be dependent on different contexts and several of the reported studies investigating selection acting on a certain trait found opposite results (Table 1). Both direct evidence of context‐dependent selection reported by specific studies (see above) and varying results reported among studies suggest that fluctuating selection may be taking place. In pied flycatchers, it seems to be a rule rather than exception that selection acting on plumage traits is variable both in time and in space. Overall, fluctuations in selection are considered the strongest known mechanism to maintain genetic variation in fitness‐related traits.

### 4.2 Signaling function in communication with conspecifics

#### 4.2.1 Signaling between conspecific males

Several suggested hypotheses for variation in coloration in the pied flycatcher males relate to dominance signaling between males, such as status signaling, signaling presence, delayed plumage maturation, delayed reproductive effort, and the conspecific female mimicry hypothesis (Table 3). Individuals displaying large and/or strikingly colored ornaments are often expected to be socially dominant. Signaling fighting ability by a visible cue may lower the costs of male–male aggression as it may reduce energy demanding attacks and territorial behavior in general among males by removing the need of fights between individuals with clear differences in resource holding potential (Rohwer, 1975, 1982; Whitfield, 1987). However, settling conflicts of interest based on variation in signaling traits requires honest signaling and that cheating (i.e., signaling high status without being able to back up the signal) is prohibited by a cost associated with production or maintaining the signal (e.g., avoid wear) (Grafen, 1990; Maynard Smith & Harper, 1988). Cheating can also be prohibited by more indirect costs of high expression of the signaling trait such as an increased predation risk or social costs where males signaling high fighting ability become more challenged by other high‐quality males or highly motivated males that want to defend their territory or female. In line with the status signaling hypothesis, variation in the dorsal plumage color of pied flycatchers may function as a reliable indicator of male fighting ability and therefore help in gaining territories and repelling intruders. Male aggressiveness and territorial behavior in relation to male black‐brown coloration have been investigated in several studies (Table 2), and dark males are generally more dominant and aggressive than brown males when possible biases in site dominance are taken into account (Järvi et al., 1987; Slagsvold & Lifjeld, 1988; Slagsvold & Sætre, 1991).

In addition to black‐brown coloration, forehead patch size has been suggested to signal male status and play a role in male–male communication and competition like in collared flycatcher (Pärt & Qvarnström, 1997). Large forehead patch size has been found to be associated with higher territorial behavior also in pied flycatchers (Table 1; Järvistö et al., 2013; Osorno et al., 2006), but some studies found no relationship between patch size and access to females or nest boxes (Dale et al., 1999; Järvistö et al., 2013). These results support status signaling hypothesis so that large forehead patch may signal a high fighting ability and likelihood to win in situations of male–male conflicts. The gained benefits of aggression do not however seem to always lead to better access to territories or females (see also mixed results from Table 1). We can conclude that both dorsal coloration and forehead patch size seem to play a role in male–male competition. One should not expect that signaling high fighting ability should always make it easier to establish a territory because signaling high fighting ability could in fact make it more difficult to establish a territory near another dominant male. This is because males are expected to bias aggression toward similar competitors (Grafen, 1990; Maynard Smith & Harper, 1988), thereby causing sexual selection through male–male competition to often lead to negative frequency‐dependent selection (Qvarnström et al., 2012).

Strong negative frequency‐dependent selection may lead to alternative male mating strategies within a population. It has been suggested that dull pied flycatcher males could benefit by mimicking conspecific females and thus avoid the costs of aggression from other male pied flycatchers (Slagsvold & Lifjeld, 1988). However, lower pairing success and even elicitation of female aggression have been proposed to be costs of female mimicry for brown males (Slagsvold & Sætre, 1991). Some support for the hypothesis has been found as in allopatry from collared flycatcher adult males tolerate brown males more than dark males (Sætre et al., 1993; Slagsvold & Sætre, 1991). Further supporting are the findings that sex‐recognition ability in the pied flycatchers is imperfect (Sætre, 1993; Slagsvold & Sætre, 1991). However, attempts to experimentally show benefits for brown males during territory establishment and holding a territory have failed (Huhta & Alatalo, 1993; Lundberg & Alatalo, 1992). Overall, the conspecific female mimicry hypothesis has gained limited support and has been mainly replaced with the heterospecific female mimicry hypothesis in the 1990s and onward (see below, Table 3). However, although brown pied flycatcher males resemble more interspecific females than conspecific females (Calhim et al., 2014), sex identification may be imperfect, and thus, the support for conspecific female mimicry hypothesis should not be completely dismissed.

#### 4.2.2 Male signaling quality to females

Female preferences for conspicuously colored males of high quality often are assumed to be a main selective pressure explaining the evolution of coloration traits in male birds (Andersson, 1994). Females are, in turn, assumed to receive benefits in terms of gained resources of superior genes by selecting males with conspicuous coloration and large ornaments as breeding partners (Andersson, 1994). Many studies have investigated whether female pied flycatcher prefer conspicuous male plumage traits (Table 1), that is, whether males with certain plumage traits have a mating advantage. Comparisons of the degree of melanin‐based coloration and 32 different fitness‐related measures from 22 different studies do, however, not reveal any clear advantage for dark males in gaining within pair or extrapair mating success (Table 1). Of 31 reported comparisons, 13 find an association between male melanin coloration and female choice. Further, eight of these 13 reported associations indicate selection for dark and one for brown coloration, while in four cases, selection is context‐dependent and varying in time (Lifjeld & Slagsvold, 1988), space (Kerimov et al., 1994), pairing status (Slagsvold & Drevon, 1999), or whether the population is allopatric or sympatric (Sætre et al., 1997). Despite several studies on female preferences, few studies have examined whether females obtain benefits, for example, in terms of higher parental effort, by selecting more conspicuous males (e.g., Järvistö et al., 2013; Sætre, 1993; Sætre et al., 1997; Slagsvold & Lifjeld, 1988). Female pied flycatchers have also been found to largely base their choice on the quality of the territory the male defend rather than on his own characteristics (Alatalo et al., 1986).

Studies on UV reflection of plumage remain few, but in three published studies, four of five measures found that female mate choice is acting on male UV reflectance. Selection for higher UV reflectance regarding male plumage in general has been detected in within pair mate choice (Siitari et al., 2002) and regarding white wing patch in extrapair mate choice (Lehtonen et al., 2009). Variation in UV reflectance has been found to have a stronger effect on pairing success in dark males than in brown ones (Sirkiä & Laaksonen, 2009). In addition, an experimental manipulation of UV reflectance revealed that females preferred males with high UV reflectance early but not late in the pairing season, suggesting time‐dependent plasticity in female choice based in UV reflectance.

For forehead patch size, only three out of 12 different measures of female mate choice in nine published papers found evidence suggesting that male forehead patch size is sexually selected in pied flycatchers. In one case, selection on forehead patch size was dependent on prevailing temperature so that the delaying effect of a cold spring on laying date was less pronounced in males with large white forehead patches than in males with small forehead patches (Sirkiä et al., 2010). In light of the existing studies, forehead patch size does not seem to be a main target for female choice in the pied flycatcher.

Even though Creutz (1955) suggested early that white patches on male wings may play important role in female choice, there are few studies that have investigated sexual selection acting on the wing patch size. Sirkiä and Laaksonen (2009) found that females preferred males with large wing patch over males with small patch. The size of the wing patch does not matter in terms of gaining extrapair copulations (Moreno, Martínez, et al., 2013; Moreno et al., 2010). In some species, brightly contrasting plumage patterns in both wings and tail are used in foraging to flush prey from their hides (Mumme, 2002), but in the pied flycatcher, such behavior has not been reported.

So far, only one study has investigated the role of tail patch size in mate choice and the trait was not found to be target for female choice (Sirkiä & Laaksonen, 2009). Interestingly, the geographical pattern in variation of tail patch size is opposite to other plumage patches: Its size is large in sympatry with collared flycatcher, and average size is decreasing with increasing distance from sympatry with collared flycatcher (Laaksonen et al., 2015). The possible adaptive function of variation in the size of the white tail patch remains unknown as the trait has been neglected in selection studies until very recent years.

Different male coloration traits are correlated with each other (Laaksonen et al., 2015), and selection on coloration traits is known to act simultaneously on several plumage traits (e.g., Sirkiä et al., 2015, Table 1), which complicates the expected evolutionary responses to selection. In addition, the relationships between coloration traits may per se be targets of selection. The use of multiple traits in mate choice seems to be common in birds (Dale, 2006). Female pied flycatchers have been observed to base their choice of male on multiple traits, including male wing patch size, UV reflectance of white wing patch, male morphological size, and song versatility simultaneously (Sirkiä & Laaksonen, 2009). Multiple ornaments may convey information about different aspects of male quality, and this information may be of different value to different females or under different conditions (Candolin, 2003). The use of multiple cues may reduce the variance in male mating success, decrease the strength of selection, and thus maintain genetic variation in male traits (Candolin, 2003).

The genic capture model offers yet another possible mechanism for maintenance of variation in male display traits. When these traits are costly to produce or maintain and hence depend upon overall condition, which itself is dependent upon genes at many loci, the expression of the sexually selected traits will capture genetic variation in all traits that influence individual condition (Rowe & Houle, 1996; Tomkins et al., 2004). The condition‐dependent nature of coloration traits makes genic capture one of the possible mechanisms maintaining variation in the face of selection in some local pied flycatcher populations. However, we consider fluctuations in selection arising from abiotic factors a more likely explanation for maintained genetic variation in coloration traits of pied flycatchers. This also means that these coloration traits not unambiguously signal individual quality across all environmental contexts. By extension, adaptive female choice should then be expected to be plastic and adjusted in accordance with the relationship between male display traits and abiotic factors when reliable cues are available (Qvarnström, 2001).

#### 4.2.3 Overall selection patterns on coloration in males

A substantial proportion of the selection studies on male plumage coloration cannot separate between potential mechanisms of sexual (i.e., function in communication with conspecific males or females) or natural selection. 11 published studies have investigated potential relationships between melanin coloration and measures of breeding success (Table 1). Among 16 reported measures of breeding success, associations between melanin coloration and breeding success were found for 12 different measures. Three of these indicated selection for dark coloration, but in most of the cases (9 breeding success measures), the detected association was dependent on the context such as space (Kerimov et al., 1994), age (Alatalo et al., 1994; Gálvan & Moreno, 2009), male breeding history (Ivankina et al., 2001), overall mean melanin coloration of males in the population (Røskaft et al., 1986), and environmental conditions (Järvistö, 2015; Sirkiä et al., 2010).

Of the four studies that have investigated the relationship between breeding success and male forehead patch size, only one study found evidence for selection favoring larger patch size (Osorno et al., 2006), one for smaller patch size (Sanz, 2001) (see Table 1). In one of the cases, selection is dependent on male age so that forehead patch size matters only for clutch size of females paired to young males (Gálvan & Moreno, 2009). Two studies that investigated the relationship between breeding success and wing patch size using long‐term data found context‐dependent selection on male coloration (see *Humidity* and *context‐dependent selection*, above).

Selection on melanin‐based dorsal coloration and forehead patch size have been quite intensively studied, but for the rest of the traits, the knowledge still remains limited. We can conclude that the pied flycatcher males with conspicuous dark plumage, large ornaments, or high UV reflectance cannot be unambiguously said to have higher fitness than males with less pronounced traits, and we are far from confident that any of the studied coloration traits would signal individual quality. Similarly, to the existing literature from studies conducted mostly in single populations, a large‐scale study examining fecundity selection on different plumage traits in 17 populations covering breeding range of the pied flycatcher did not find evidence that there would be constant selection for conspicuous plumage in allopatry (Sirkiä et al., 2015).

It is important to take into consideration that most of the data sets used in the selection studies are to some extent biased toward successful males, as it is difficult to get information of the males that do not manage to pair at all or if the breeding attempts fail before the male was captured (Both et al., 2017). The knowledge of the proportion of nonbreeding males is very scarce (but see Sternberg, 1989; Sternberg et al., 2002), and selection acting before pairing may differ between populations. This means that the role of variation in male coloration both for the establishment of breeding territories and for attracting a female to breed with may be underestimated in these studies.

### 4.3 Signaling function in communication with heterospecifics

The hypotheses for existence of male color variation in male pied flycatchers that have gained most attention are perhaps the ones related to interspecific interactions with the collared flycatcher. The distributions of the pied flycatcher and the closely related collared flycatcher (*Ficedula albicollis*) overlap in Central and Eastern Europe (Lundberg & Alatalo, 1992; Figure 1; Cramp & Simmons, 2006). These two species diverged during the Pleistocene glaciations less than a million years ago (Nadachowska‐Brzyska et al., 2013), and have probably gone through cycles of geographical isolation in separate refugia of the Mediterranean area during the ice ages followed by breeding range expansions northward. There are two contact zones; one broad hybrid zone in Central and Eastern Europe, and one younger and more isolated hybrid zone on the Baltic islands of Öland and Gotland, Sweden (Lundberg & Alatalo, 1992; Qvarnström et al., 2010). There is only a slight temporal difference in times of breeding (Alatalo et al., 1990; Qvarnström et al., 2009; Sætre et al., 1999; Sirkiä et al., 2018), and little divergence in size (Merilä et al., 1994), in feeding techniques (Alerstam et al., 1978), or in the diet (Wiley et al., 2007). Moreover, both species breed in nest cavities (or nest boxes when provided) in deciduous forest leading to competition where collared flycatchers are more dominant (Qvarnström et al., 2010; Sætre & Sæther, 2010) and replacing the pied flycatcher from the most preferred habitats (Rybinski et al., 2016). Interspecific relationships between the pied and collared flycatcher have been studied extensively. There is evidence of character displacement in the pied flycatcher in the sympatric area with respect to ecological, social, and sexual traits (reviewed by Qvarnström et al., 2010; Sætre & Sæther, 2010), and pied males in sympatry express mostly dull brown coloration, with low UV reflectance, small forehead and wing patches, and large tail patch (Laaksonen et al., 2015).

#### 4.3.1 Avoidance of hybridization

Avoidance of hybridization with collared flycatcher has been suggested to be the main cause for divergence in male plumage coloration in the pied flycatcher in sympatry. Female mate preferences are species‐assortative in both pied and collared flycatchers (Sætre et al., 1997), and collared flycatcher females paired with heterospecific males tend to have extrapair copulations with conspecific males (Veen et al., 2001). Hybridization has high costs as female and male hybrids are sterile, and hybrid males have very low fitness (Ålund et al., 2013; Svedin et al., 2008). Avoidance of hybridization is thus beneficial and brown males are favoured in mate choice by conspecific females in sympatry (Sætre et al., 1993, 1997), which supports the hypothesis that dull plumage would be an adaptation to avoid hybridization. However, while dull brown males are able to establish territories closer to collared flycatchers (Alatalo et al., 1994; Vallin et al., 2012), as a side effect of being more likely to breed closer to collared flycatchers, brown males experience higher risk of hybridization under natural conditions (Vallin et al., 2012). Thus, opposite to the hypothesis dull males have actually higher risk to end up paired with a collared flycatcher female and suffer from extremely low fitness. These findings weaken the support for avoidance of hybridization being the main mechanism to drive to plumage divergence in sympatric zone.

#### 4.3.2 Heterospecific female mimicry

Another closely related hypothesis for plumage diverge in sympatry is that brown male pied coloration is an adaptation to avoid aggression and competition from heterospecific males. It is known that dull coloration reduces interspecific male–male aggression (Sætre et al., 1993) and brown male pied flycatchers are allowed to settle closer to resident male collared flycatchers than black male pied flycatchers (Alatalo et al., 1994; Vallin et al., 2012). Brown males have relatively higher breeding success than black males in woodlots where collared flycatchers are present likely due to reduced aggression from collared flycatchers (Vallin et al., 2012). A recent study shows that the brown male phenotype of the pied flycatchers mimics heterospecific females rather than intraspecific females (Calhim et al., 2014) further supporting the view that the brown phenotype is an adaptation to avoid interspecific male aggression. However, while brown males benefit from avoiding heterospecific competition and have higher relative fitness than black males when co‐occurring with collared flycatchers, those simultaneously have higher risk of hybridization (Vallin et al., 2012). Competition between heterospecific males can be hence considered the main driving force leading to fast reproductive character displacement in sympatry. However, these findings do not rule out the possibility of reinforcement acting in parallel at a slower rate. In the old Central Europe hybrid zone, the pied flycatcher females have indeed been found to prefer brown males over black ones (Sætre et al., 1997), which should reduce the risk of making mate choice errors. Risks of hybridization or heterospecific female mimicry have not been studied in relation to other plumage traits than black‐brown coloration, but there is broad expectation that all intercorrelated plumage traits have been selected by the same processes and evolved together.

#### 4.3.3 Interplay between interspecific relationships and environmental conditions

Interspecific relationships seem to interplay with environmental conditions, which may play a role in the maintenance of color variation in the pied flycatcher males. Differences in the overall breeding ranges of the pied and collared flycatchers in Europe imply that collared flycatchers are relatively more limited by climate. In addition, in the large Central European contact zone collared flycatchers are numerously dominant in warmer lowland areas, whereas the pied flycatchers are more common in colder boreal and subalpine zones (Sætre et al., 1999; Sætre, Post, et al., 1999). The relationship between climate tolerance and aggressive behavior/dominance signaling appears to differ between the two flycatcher species. The suggested most dominant, black pied flycatchers with large forehead patches appear relatively better adapted to northern climate with cold spring temperatures than brown males with smaller patches (Järvistö, 2015; Sirkiä et al., 2010, 2013), while the most dominant collared flycatchers, that is, with large forehead patch sizes, instead are worse adapted to northern climate conditions than males with small forehead patches (Robinson et al., 2012). Divergence in plumage traits and suggested dominance signaling is therefore associated with convergence in climate requirements. It has been suggested that life history adaptations and sexually selected traits coevolve in the two flycatcher species and that these evolutionary processes have been affected by periods of repeated glaciations and interglacials during speciation (Qvarnström et al., 2016).

### 4.4 Different selection regimes and gene flow maintaining color variation among populations

Population differentiation in phenotypic traits is expected to reflect a balance between the diversifying effect of local, spatially variable selection and the homogenizing effect of gene flow (Endler, 1980; Kirkpatrick & Barton, 1997). In the pied flycatcher, the conspicuousness in male plumage traits increases in allopatry in relation to the distance to the Central European sympatric area (Laaksonen et al., 2015). For black‐brown coloration, the pattern has been described relatively early (Huhta & Siikamäki, 1997; Lehtonen, Laaksonen, et al., 2009; Lundberg & Alatalo, 1992; Røskaft & Järvi, 1992), and often, the increasing conspicuousness in plumage traits in relation to the distance from the sympatric area has been assumed to be caused by a combination of selection for less conspicuous coloration in symparic area and selection for more conspicuous male plumage coloration in allopatric areas.

Large‐scale studies show that there is much more phenotypic variation in the plumage traits of male pied flycatchers among populations than predicted by neutral genetic variation (Laaksonen et al., 2015; Lehtonen, Laaksonen, et al., 2009). Such patterns are commonly interpreted as an indirect signal of divergent selection on a trait (Leinonen et al., 2008), which supports the hypothesis that there is selection for conspicuous plumage coloration in allopatry. There is selection for dull brown coloration and small ornament sizes mimicking collared females at least in old Central European hybrid zone (see above). However, the situation seems to be more complicated in allopatry. As we summarize above, despite extensive research effort there is no consensus in the literature that conspicuous male traits would always be selected for (see above, Table 1). While gene flow and dispersal in the pied flycatcher remain relatively poorly understood, the genetic population structure indicates that populations breeding in northern Europe appear to be panmictic (Lehtonen, Laaksonen, et al., 2009). Long‐term study comparing phenotypic variation in black‐brown dorsal coloration of male pied flycatchers supports the hypothesis that gene flow from sympatric areas with collared flycatcher is maintaining phenotypic variation among populations in allopatry (Sirkiä et al., 2013). We however miss the information if different male color types have different long‐distance dispersal propensities. Dispersal propensity of different male phenotypes is rather interesting question in light of current environmental changes and needs to rapidly adapt to changing climate.

## 5 CONCLUSIONS

The striking variation in plumage coloration of male pied flycatchers, especially the dorsal breeding coloration ranging from dull brown to shiny black, has gained a lot of scientific attention. Numerous studies have investigated the proximate determination and signaling function of various plumage coloration traits and have tried to explain the persistence of variation in these traits (Tables 1, 2, 3). A meta‐analyses based on research performed on the signaling role of coloration in the blue tit concluded few solid findings and a lack of replication of main findings (Parker, 2013). In this review, we have only summarized previous findings, but it is also in the flycatcher case clear that some conclusions are more solid than others and that replicated studies often report conflicting results. While caution is needed with respect to publication biases (we expect replicated studies to be easier to publish if the results are different from previous findings), a main take‐home message is that selection patterns acting on signaling traits are more diverse and fluctuating than generally expected.

**FIGURE 3:**
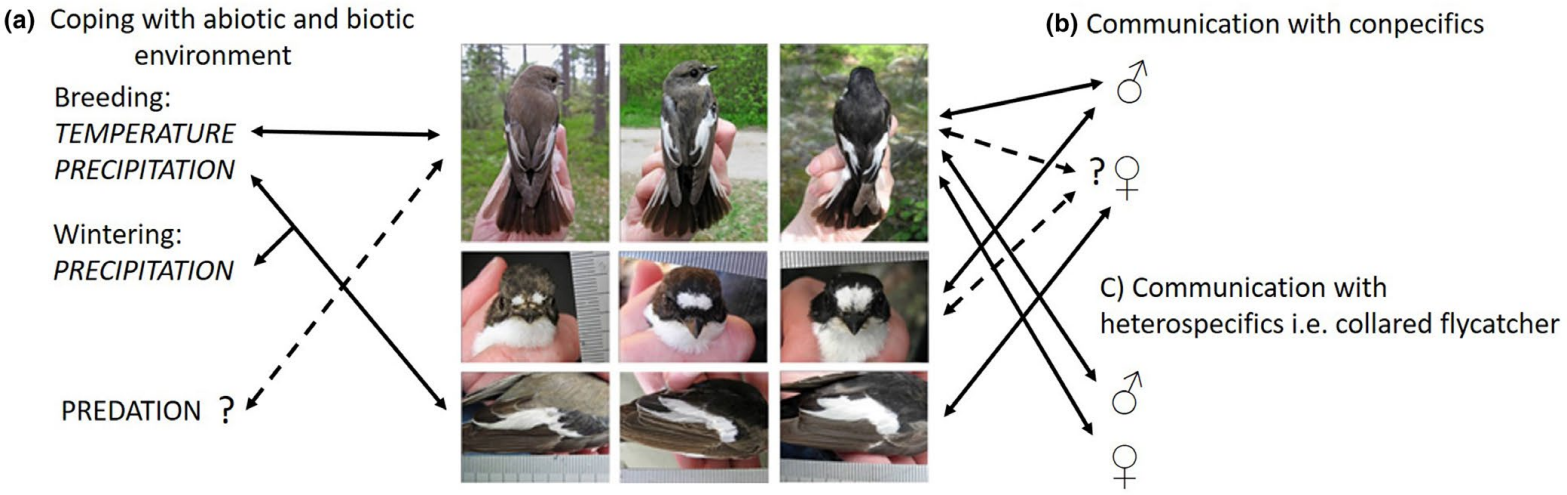
Adaptive functions of male coloration traits in pied flycatchers. Males vary in coloration to (a) cope with variation in the biotic and abiotic environment, (b) communicate with conspecific males or females, and (c) communicate with heterospecifics. The typical northern climate with cold springs but relatively high summer temperature favors dark dorsal coloration. Studies testing the role of male melanin coloration as antipredator strategy have, however, reported conflicting results. Dark coloration is also associated with dominance in male–male competition among conspecifics. However, brown males are favored in competition with dominant collared flycatchers in areas of sympatry. Female choice also favors brown males in sympatry but is more variable in allopatry than originally thought. Female choice may instead “track” how well different colored males do under various weather conditions and relation to the frequency of male phenotypes in the population. In general, large forehead and wing patches covary with dark dorsal coloration and seem to have similar functions in relation to abiotic environment and communication. Male forehead patch size functions as a dominance signal in male–male competition. However, different plumage traits should not be expected to signal high quality as such but rather different male competitive strategies. Selection on wing patch size has been found to be dependent on the amount of precipitation during both breeding season and wintering conditions. Nestlings of males with large wing patches have lower mortality in years with high levels of rainfall. Further, after experiencing a relatively dry winter, large‐patched males were more successful in attracting females that laid large clutches and were more likely to survive. While results of tens of female choice studies show that males with relatively conspicuous plumage traits are not unambiguously preferred by females, female choice favors both males with a large wing patch and males with high UV reflection on their wing patch

The suggested adaptive functions of variation in coloration traits of male pied flycatchers can be sorted into three nonmutually exclusive main categories. Males may vary in coloration to (1) cope with variation in the biotic and abiotic environment, (2) communicate with conspecifics, and (3) communicate with heterospecifics (Figure 3). First, pied flycatchers have a large geographical breeding range and the various color phenotypes have been suggested to be adaptations to cope with altering temperature, humidity, habitat, predation and parasitism levels. Several studies have indeed supported the hypothesis that different melanin color types are adapted to different prevailing temperatures.

Second, hypotheses for adaptive function of coloration relate to signaling both to males and to females. Overall, there is fairly strong evidence that dark plumage color function as a dominance signal in male–male competition and across species dark melanic individuals are usually more dominant than lighter ones (see, e.g., Ducrest et al., 2008). Signaling high dominance may not always translate into an advantage in gaining access to resources or females, especially not in areas of co‐occurrence with the more aggressive collared flycatcher.

There is strong evidence that interspecific interactions with collared flycatchers are of crucial importance for explaining the origin and maintenance of plumage color variation in male pied flycatchers. However, recent findings have modified the traditional views of the effects of heterospecific relationships on plumage coloration. Intersexual competition with male collared flycatchers seems to play a more central role in driving character displacement in plumage divergence, while the role of avoidance of hybridization may not be as important as has been thought earlier. While hybridization is very costly for the individuals involved, the territorial interactions with heterospecific males are simply much more frequent than the interactions with heterospecific females and therefore have a larger impact on the patterns observed under natural conditions. Although selection patterns acting in sympatry with collared flycatchers may be reflected across the whole distribution area due to dispersal and gene flow, selection regimes are not simply acting in opposite direction in sympatry and allopatry as traditionally was expected. Selection patterns acting on variation in plumage coloration of pied flycatchers are more variable and context‐dependent in allopatry than previously thought.

We can conclude that several mechanisms contribute to the maintenance of variation in male plumage coloration in the pied flycatcher. These mechanisms include different selection regimes acting in sympatry or allopatry with the closely related collared flycatchers accompanied by gene flow across the whole breeding range due to dispersal from the hybrid zones, as well as fluctuating selection in time and space also in the absence of collared flycatchers. Future studies are hopefully also better able to take carry‐over effects from environmental conditions experienced on the wintering grounds and the whole flyway into account.

The current movement toward open data access is promising in terms of both increased quality and speed of scientific progress, but there is an increasing risk that the responsibility for rigorous data collection and experimental design become detached from downstream scientific efforts (Mills et al., 2015). Detailed observations of how animals actually behave in their natural environments are critical for our understanding patterns in the data because the devil may be hidden in the details. The number of long‐term ecological studies, such as many of the studies reviewed in this paper, is strongly declining during the 21st century. At the same time, new population monitoring studies are rarely initiated and often have severe difficulties in terms of continuous funding. While there is a tendency to rely on short‐term data collection efforts or data collected by others, we want to highlight the importance of behavioral and long‐term studies in resolving wide and complicated questions related to, for example, wild population ability to adjust to fast environmental changes in terms of habitat loss and climate change.

## CONFLICT OF INTEREST

The authors are unaware of any conflicts of interest.

## AUTHOR CONTRIBUTIONS

Päivi M. Sirkiä: Conceptualization (lead); funding acquisition (lead); methodology (lead); project administration (lead); visualization (lead); writing—original draft (lead); writing—review & editing (equal). Anna Qvarnström: Conceptualization (supporting); methodology (supporting); visualization (supporting); writing—original draft (supporting); writing—review and editing (supporting).

## Supporting information

Supplementary Material

## Data Availability

This paper is review, and results are not based on any new data that could be archived.
