# Supplementary material for: Adaptive coloration in pied flycatchers (Ficedula hypoleuca)—The devil is in the detail
Source: Ecol Evol. 2021 Jan 24;11(4):1501–25. doi: 10.1002/ece3.7048 (PMC7882974; doi:10.1002/ece3.7048)
Supplement: Supplementary file 1 — Supplementary Material [file ECE3-11-1501-s001.docx]

**Supplementary information**

*Additional suggested hypotheses that may play a role in persistence of colour variation*

Frequency- and density-dependent selections could provide new and more accurate views on spatial patterns of selection on plumage colouration and on net selection acting on phenotypes. Frequency-dependent selection is one of the major mechanisms that can maintain variation in fitness related traits (Smith 1982) and it could act for example via predation if the predators preferentially attack the most frequent colour types. Potential negative frequency-dependent effects in male-male competition driven by different colour phenotypes with different strategies (Qvarnström et al. 2012) should be investigated. While intra- and interspecific density-dependent selection on colouration traits remains unclear, one has to keep in mind that most of the studies examining male colouration traits and fitness components have been done in relatively dense nest-box areas in which the densities may differ from prevailing forests mostly without nest-boxes.

In addition to mechanisms reviewed above, other selection pathways, such as sexually antagonistic selection could provide new and more accurate views on selection acting on plumage traits. While sexually antagonistic selection remains unexplored in the pied flycatcher, the scientific interest in female colouration traits has been recently increasing. Females have been found to vary in several plumage traits: dorsal brown colouration and mantle colouration (Potti et al. 2014), size of the white wing patch (Morales et al. 2007) and even forehead patch that some females express in the Iberian population (Potti 1993). Female wing patch size has been observed to be associated with physiology (Lopez-Arrabe et al. 2014, Moreno et al. 2013b, Moreno et al. 2014, Morales et al. 2007) and breeding success (Morales et al. 2007). The expression of a forehead patch in Iberian population is associated with age (Potti and Merino 1995), blood parasitism (but see Potti and Merino 1996, Morales et al. 2007), physiology (Lopez-Arrabe et al. 2014, Moreno et al. 2013b) and fledging success, lifetime fecundity and survival (Potti et al. 2013). While adaptive functions of female colouration traits remain unresolved forehead patch has been suggested to play a role in intrasexual competition between females (Morales et al. 2014). Also assortative pairing in relation to colouration traits has gained only little attention, but there is some indication of assortative pairing in relation to wing and forehead patches (Moreno et al. 2013a, but see Lopez-Arrabe et al. 2014).

While mortality based selection on melanin-based colouration has been found in barn owls (Roulin et al. 2010), tawny owls (Karell et al. 2011), barn swallows (Saino et al. 2013) and urban feral pigeons (Récapet et al. 2013), studies on the relationship between return rate and male colouration traits in the pied flycatchers have yielded conflicting results (Table 1). Both brown (Røskaft et al. 1986, Järvi et al. 1987) and black (Potti and Montalvo 1991, Belskii and Lyakhov 2004) males have found to have higher return rates but most of the studies have not detected any such relationship (Lundberg and Alatalo 1992, Slagsvold and Lifjeld 1988, Alatalo et al. 1994, Ivankina et al. 2001). Compared to pairing and breeding success, survival of different colour phenotypes has gained little attention, foremost because the ability to distinguish between survival and dispersal is limited. In addition, we are still lacking studies that would take environmental conditions in the whole flyway; in the breeding grounds, in wintering areas and on migration, into account. Similarly, good estimates of recruitment rates and lifetime reproductive success estimates are very scarce due to dispersal and limited methods of tracking movements of small passerines, which limits the conclusion made on the role for persistence of colour variation. Recent development of technologies provides better methods to follow the movements and will likely facilitate understanding role of dispersal strategies of differently coloured males.

Studies on plumage colouration traits and fitness have focused almost solely on directional selection, but selection on male plumage colouration may also be non-linear: stabilizing, disruptive or frequency dependent. Overall, one has to bear in mind that maintenance of variation among populations may consist of different combinations of directional and non-linear selection. It should also be kept in mind that any description of spatial patterns of selection is a snapshot of temporal dynamics of selection (Siepielski et al. 2009, Morrissey and Hadfield 2012). Thus, in order to deepen our understanding of phenotypic selection in free-living populations, spatial and temporal aspects of selection should be integrated in future studies.

ALATALO, R. V., GUSTAFSSON, L. and LUNDBERG, A. 1994. Male coloration and species recognition in sympatric flycatchers. -*Proceedings of the Royal Society of London Series B-Biological Sciences,* **256**: 113-118.

BELSKII, E. A. and LYAKHOV, A. G. 2004. Breeding plumage colouration of males Ficedula hypoleuca Pall. (Passeriformes, Muscicapidae) in the Middle Urals. -*Zoologicheskiy zhurnal,* **83**: 1468-1475.

IVANKINA, E. V., GRINKOV, V. G. and KERIMOV, A. B. 2001. Male colour type and lifetime breeding success in the Pied Flycatcher Ficedula hypoleuca. -*Acta Ornithologica,* **36**: 91-96.

JÄRVI, T., RØSKAFT, E., BAKKEN, M. and ZUMSTEG, B. 1987. Evolution of variation in male secondary sexual characteristics - a test of eight hypoheses applied to pied flycatchers. -*Behavioral Ecology and Sociobiology,* **20**: 161-169.

KARELL, P., AHOLA, K., KARSTINEN, T., VALKAMA, J. and BROMMER, J. E. 2011. Climate change drives microevolution in a wild bird. -*Nature Communications,* **2**: 1-7.

LOPEZ-ARRABE, J., CANTARERO, A., PEREZ-RODRIGUEZ, L., PALMA, A. and MORENO, J. 2014. Plumage ornaments and reproductive investment in relation to oxidative status in the Iberian Pied Flycatcher (Ficedula hypoleuca iberiae). -*Canadian Journal of Zoology,* **92**: 1019-1027.

LUNDBERG, A. and ALATALO, R. V. 1992. *The pied flycatcher,* London, T & AD Poyser.

MORALES, J., GORDO, O., LOBATO, E., IPPI, S., MARTINEZ-DE LA PUENTE, J., TOMAS, G., MERINO, S. and MORENO, J. 2014. Female-female competition is influenced by forehead patch expression in pied flycatcher females. -*Behavioral Ecology and Sociobiology,* **68**: 1195-1204.

MORALES, J., MORENO, J., MERINO, S., SANZ, J. J., TOMAS, G., ARRIERO, E., LOBATO, E. and DE LA PUENTE, J. M. 2007. Female ornaments in the Pied Flycatcher Ficedula hypoleuca: associations with age, health and reproductive success. -*Ibis,* **149**: 245-254.

MORENO, J., GIL, D., CANTARERO, A. and LOPEZ-ARRABE, J. 2014. Extent of a white plumage patch covaries with testosterone levels in female Pied Flycatchers Ficedula hypoleuca. -*Journal of Ornithology,* **155**: 639-648.

MORENO, J., VELANDO, A., GONZÁLEZ-BRAOJOS, S., RUIZ-DE-CASTAÑEDA, R. and CANTARERO, A. 2013a. Females Paired with More Attractive Males Show Reduced Oxidative Damage: Possible Direct Benefits of Mate Choice in Pied Flycatchers. -*Ethology,* **119**: 727-737.

MORENO, J., VELANDO, A., RUIZ-DE-CASTANEDA, R., GONZALEZ-BRAOJOS, S. and CANTARERO, A. 2013b. Oxidative damage in relation to a female plumage badge: evidence for signalling costs. -*Acta Ethologica,* **16**: 65-75.

MORRISSEY, M. B. and HADFIELD, J. D. 2012. Directional selection in temporally replicated studies is remarkably consistent. -*Evolution,* **66**: 435-442.

POTTI, J. 1993. A MALE TRAIT EXPRESSED IN FEMALE PIED FLYCATCHERS, FICEDULA-HYPOLEUCA - THE WHITE FOREHEAD PATCH. -*Animal Behaviour,* **45**: 1245-1247.

POTTI, J., CANAL, D. and CAMACHO, C. 2014. Ontogenetic variation in the plumage colour of female European Pied Flycatchers Ficedula hypoleuca. -*Ibis,* **156**: 879-884.

POTTI, J. and MERINO, S. 1995. SOME MALE PIED FLYCATCHERS FICEDULA-HYPOLEUCA IN IBERIA BECOME COLLARED WITH AGE. -*Ibis,* **137**: 405-409.

POTTI, J. and MERINO, S. 1996. Decreased levels of blood trypanosome infection correlate with female expression of a male secondary sexual trait: Implications for sexual selection. -*Proceedings of the Royal Society B-Biological Sciences,* **263**: 1199-1204.

POTTI, J. and MONTALVO, S. 1991. Male arrival and female mate choice in pied flycatchers Ficedula hypoleuca in Central Spain. -*Ornis Scandinavica,* **22**: 45-54.

QVARNSTRÖM, A., VALLIN, N. and RUDH, A. 2012. The role of male contest competition over mates in speciation. -*Current Zoology,* **58**: 493-509.

RÉCAPET, C., DAUPHIN, L., JACQUIN, L., GASPARINI, J. and PRÉVOT-JULLIARD, A. C. 2013. Eumelanin-based colouration reflects local survival of juvenile feral pigeons in an urban pigeon house. -*Journal of Avian Biology,* **44**: 583-590.

ROULIN, A., ALTWEGG, R., JENSEN, H., STEINSLAND, I. and SCHAUB, M. 2010. Sex-dependent selection on an autosomal melanic female ornament promotes the evolution of sex ratio bias. -*Ecology Letters,* **13**: 616-626.

RØSKAFT, E., JÄRVI, T., NYHOLM, N. E. I., VIROLAINEN, M., WINKEL, W. and ZANG, H. 1986. Geographic variation in secondary sexual plumage colour characteristics of the male pied flycatcher. -*Ornis Scandinavica,* **17**: 293-298.

SAINO, N., ROMANO, M., RUBOLINI, D., AMBROSINI, R., CAPRIOLI, M., MILZANI, A., COSTANZO, A., COLOMBO, G., CANOVA, L. and WAKAMATSU, K. 2013. Viability is associated with melanin-based coloration in the barn swallow (Hirundo rustica). -*Plos One,* **8**: 6.

SIEPIELSKI, A. M., DIBATTISTA, J. D. and CARLSON, S. M. 2009. It's about time: the temporal dynamics of phenotypic selection in the wild. -*Ecology Letters,* **12**: 1261-1276.

SLAGSVOLD, T. and LIFJELD, J. T. 1988. Plumage color and sexual selection in the pied flycatcher Ficedula hypoleuca. -*Animal Behaviour,* **36**: 395-407.

SMITH, J. M. 1982. *Evolution and the Theory of Games*, Cambridge University Press.
